# Study on the Relationship between Electron Transfer and Electrical Properties of XLPE/Modification SR under Polarity Reversal

**DOI:** 10.3390/polym16162356

**Published:** 2024-08-20

**Authors:** Zhi-Yuan Wu, Yu-Zhi Jin, Zhe-Xu Shi, Zhi-Yuan Wang, Wei Wang

**Affiliations:** 1 School of Electrical and Electronic Engineering, North China Electric Power University, Beijing 102206, China; 2 Northwest Branch of State Grid Corporation of China, Xi’an 710199, China

**Keywords:** polarity reversal, nanoparticle composition, XLPE/SR, space charge distribution, breakdown characteristics

## Abstract

The insulation of high-voltage direct-current (HVDC) cables experiences a short period of voltage polarity reversal when the power flow is adjusted, leading to sever field distortion in this situation. Consequently, improving the insulation performance of the composite insulation structure in these cables has become an urgent challenge. In this paper, SiC-SR (silicone rubber) and TiO_2_-SR nanocomposites were chosen for fabricating HVDC cable accessories. These nanocomposites were prepared using the solution blending method, and an electro-acoustic pulse (PEA) space charge test platform was established to explore the electron transfer mechanism. The space charge characteristics and field strength distribution of a double-layer dielectric composed of cross-linked polyethylene (XLPE) and nano-composite SR at different concentrations were studied during voltage polarity reversal. Additionally, a self-built breakdown platform for flake samples was established to explore the effect of the nanoparticle doping concentration on the breakdown field strength of double-layer composite media under polarity reversal. Therefore, a correlation was established between the micro electron transfer process and the macro electrical properties of polymers (XLPE/SR). The results show that optimal concentrations of nano-SiC and TiO_2_ particles introduce deep traps in the SR matrix, significantly inhibiting charge accumulation and electric field distortion at the interface, thereby effectively improving the dielectric strength of the double-layer polymers (XLPE/SR).

## 1. Introduction

High-voltage direct-current (HVDC) transmission enables long-distance, high-capacity power transmission, facilitating cross-regional interconnection of large power grids [1,2,3,4]. It is an important technology addressing the energy consumption challenge in long-distance transmission. Currently, the main insulation material used for HVDC cables is XLPE (cross-linked polyethylene). In real operation environments, high-voltage power transmission involves not only cables but also numerous cable accessories. These cable accessories are crucial for connecting different cable sections, ensuring both the extension of the cable length and the reliability of insulation at the connection. The insulation accessories are mainly made from rubber, such as silicone rubber (SR) and ethylene propylene diene monomer (EPDM).

Currently, HVDC transmissions experience power flow reversal, which is managed using line-commutated converters (LCC-HVDC). During the reversal period, this transmission method generates local transient electric fields with extremely high field strength at cable and cable accessory connections, which may exceed the insulation limits of the materials and lead to destructive damage to the DC cables. Therefore, the insulation properties of a cable under polarity reversal conditions are more important than those under DC electric fields [5,6,7]. The accumulation of space charge during operation is commonly identified as a key factor contributing to insulation failure.

Current research on space charge accumulation mainly focuses on the M-W polarization theory. Wang Xia et al. from Xi’an Jiaotong University [8] and Lan Li et al. from Shanghai Jiaotong University [9] measured the distribution of space charge and interface charge in double-layer media under different field strengths. In their study, double-layer structures composed of XLPE/SR and XLPE/ethylene propylene rubber (EPR) were selected, respectively. The experimental results indicate that the polarity of the interface charge is consistent with the polarity of the accumulated charge on the SR or EPR side under low field strength, but at high field strength (about 30 kV/mm or above), the polarity of the interface charge reverses and deviates significantly from the theoretical calculation value. At the same time, there have been many studies on the effect of nanoparticle-doping modification on the space charge distribution characteristics. Yang Zhuoran et al. from Tianjin University [10] also studied the effect of the doping SiC concentration on the resistivity, space charge accumulation, and dissipation rules of SR composite insulation media. Their conclusions show that this method can indeed improve the nonlinear conductivity of silicone rubber composite materials, facilitate the dissipation of space charges, and improve the accumulation and distortion of space charges and electric fields. Qin Jun and others from the Harbin Institute of Technology [11] tested the distribution of space charges in nano-TiO_2_/LSR composite materials, and the results showed that the introduction of nano-TiO_2_ in the composite material inhibited the accumulation of homopolar charges.

However, there is relatively little research on the effect of nano-doping on the space charge of bio-layer polymers under polarity reversal. Chen Xi et al. [12] analyzed the changes in space charge characteristics and maximum transient electric field in low-density polyethylene (LDPE) samples under different polarity reversal times by controlling the voltage polarity reversal time. The experiment found that the maximum transient electric field occurred near the anode during the polarity reversal process, and the maximum transient electric field decreased with increasing reversal time. Dissado L.A. et al. from the University of Leicester in the UK analyzed the location of the maximum field strength of the cable body during the polarity reversal process and compared the space charge distribution before and after the polarity reversal under steady-state conditions. They found that the charge distribution waveform would basically form a charge peak with the same amplitude and opposite polarity at the same position, presenting a “mirror distribution”. Nevertheless, the accumulation of space charges is closely related to the breakdown characteristics. Xiang Min from Chongqing University [13,14] also investigated the corresponding breakdown characteristics of oil-paper insulation structures under the conditions of polarity reversal after DC preloading. The conclusion drawn was that during the polarity reversal process, multiple factors, such as the charge inside the insulation paper, interface polarization charge, and capacitance distribution electric field, have a certain impact on the breakdown characteristics of oil-paper insulation.

Unfortunately, previous research has only explored the space charge and breakdown characteristics of individual composite materials under polarity reversal. Research on the double-layer XLPE/SR composite insulation structure under polarity reversal conditions, which is reflective of actual operation, is still lacking, especially regarding the mechanism of insulation breakdown induced by electric field distortion caused by changes in interface charge accumulation. Such studies are urgently needed for the industrial manufacturing of cable accessories. This article establishes a PEA space charge measurement platform capable of applying a polarity reversal electric field and a breakdown test platform for thin film sheet insulation samples. SiC-SR and TiO_2_-SR nanocomposites with different doping concentrations were prepared. In this paper, we tested the distribution of space charges in XLPE/nano-doped SR bilayer media under polarity reversal. We built a correlation between the micro-level charge distribution characteristics and macro-level breakdown insulation characteristics of the bilayer composite materials. Additionally, the mechanism of electric field distortion-induced insulation breakdown due to charge accumulation was systematically studied.

## 2. Sample Preparation and Experimental Methods

### 2.1. Preparation of Nano-Doped SiC-SR and TiO_2_-SR Composite Materials

**Figure 1:**
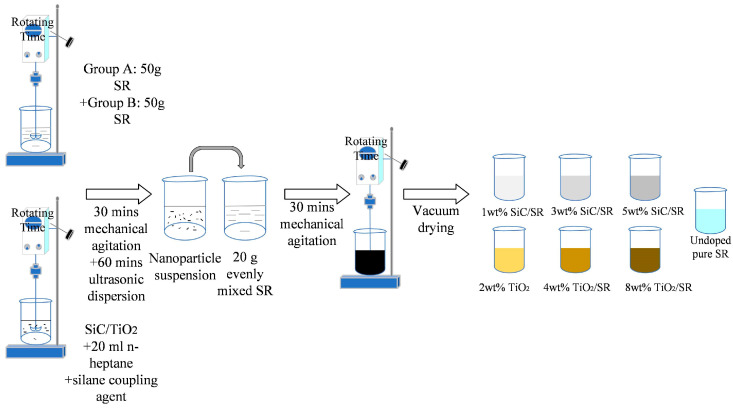
Preparation process diagram of nano-doped composite materials.

We selected the solution blending method for this study. A mechanical stirring method was used to treat the n-hexane solution containing nano-SiC and TiO_2_, with an appropriate amount of silane coupling agent added. Next, the suspension of SiC and TiO_2_ was subjected to an ultrasonic dispersion method for further treatment. Finally, the obtained nano-SiC and TiO_2_ suspension was thoroughly mixed with the liquid silicone rubber matrix to produce nano-SiC-SR and nano-TiO_2_-SR composite materials. SiC-SR composite materials with mass fractions of 1 wt%, 3 wt%, and 5 wt%, as well as TiO_2_−SR composite materials and undoped pure SR composite materials with mass fractions of 2 wt%, 4 wt%, and 8 wt%, were produced. The preparation flow chart is shown in Figure 1. The ultrasonic dispersion meter was made in Shanghai China, the double plate vulcanizer was made in Guangdong China.

### 2.2. Preparation of Sheet-like Specimens of XLPE and SiC-SR, TiO_2_-SR Nanocomposites

#### 2.2.1. Preparation of XLPE Sheet-like Specimens

**Figure 2:**
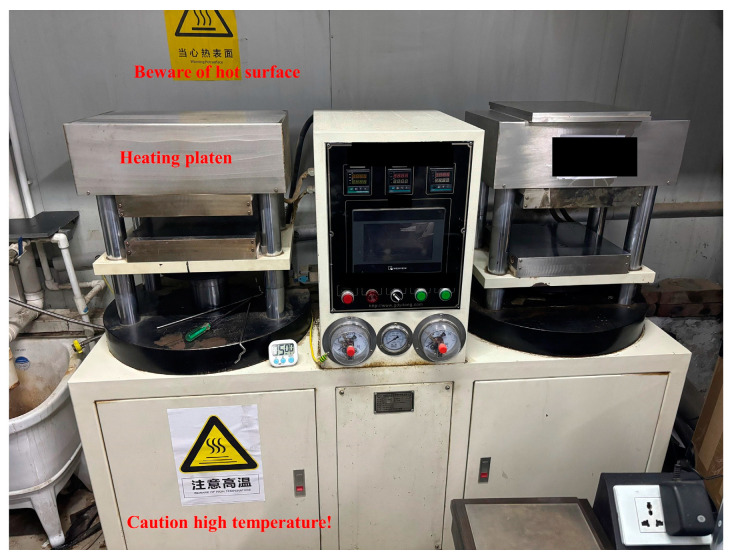
Flat vulcanizing machine.

**Figure 3:**
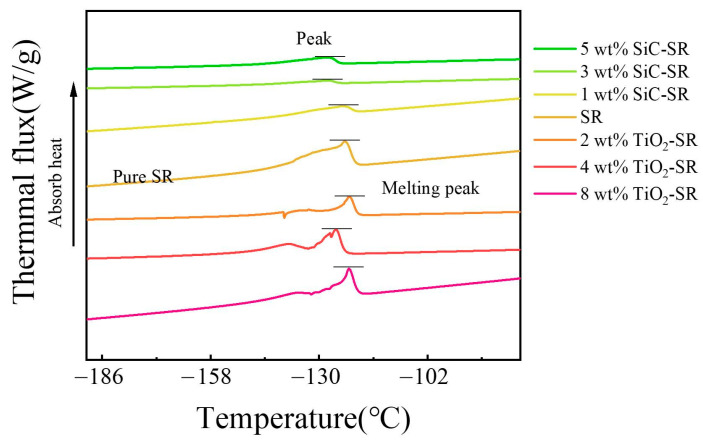
DSC curve of different SiC-SR/TiO_2_-SR samples.

**Figure 4:**
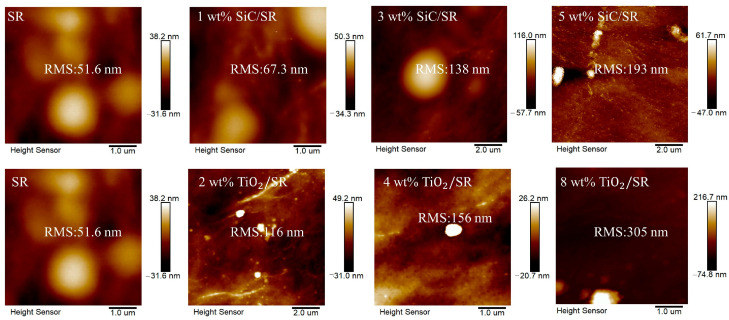
AFM of different SiC-SR/TiO_2_-SR samples.

Weigh the appropriate quality of XLPE pellets and place them on a mold lined with polyethylene film. Set the temperature of the vulcanization side of the flat vulcanization machine (Figure 2) to 145 °C and maintain a pre-heating pressure of 10 MPa for 7 min. Once the solid particles have melted, increase the temperature to 180 °C and the pressure to 14 MPa, and maintain these conditions for 9 min. Then, transfer the mold to the cooling side and allow it to cool for 5 min. Considering that the SR insulation in actual cable joint structures is usually thicker than the main insulation XLPE, and taking account the experimental conditions of breakdown tests and PEA tests, the breakdown samples were prepared by adjusting the mass of XLPE particles and the pressure between the upper and lower plates. The required size for PEA samples is 50 mm × 50 mm × 0.22 mm sheet samples, and the required size for breakdown tests is 50 mm × 50 mm × 0.02 mm sheet samples. The Zeta potential/DSC/AFM of the samples are shown in Table 1 and Figure 3 and Figure 4. We can see that the dispersion of the particles in the colloid is good, the nano-doping will decrease the melting peak of SR, and the roughness in 5 wt% SiC/SR is 193 nm and the roughness in 8 wt%TiO_2_/SR is 305 nm, a large increase compared with pure SR, which is 51.6 nm.

#### 2.2.2. Preparation of SiC-SR and TiO_2_-SR Sheet-like Specimens

Evenly apply an appropriate amount of vacuum-dried nano-doped SR material onto the mold covered with a polyimide film. Firstly, maintain the temperature at 140 °C and the interpolate pressure at 6 MPa, heating for 8 min. Then, continue heating for an additional 9–10 min at 145 °C and 10 MPa. For high-concentration doped SR samples, the temperature, pressure, and duration should be adjusted accordingly to ensure the full vulcanization of the sheet-like samples. Afterwards, the samples should undergo the same cooling treatment as previously described. Two types of specimens were prepared, one with dimensions of 50 mm × 50 mm × 0.3 mm for PEA testing, and another with dimensions of 50 mm × 50 mm × 0.08 mm for breakdown testing. Before conducting the experiment, all prepared samples should be placed in a vacuum drying oven at 40 °C for 24 h.

### 2.3. Polarity Reversal PEA Testing Platform and Testing Methods

**Figure 5:**
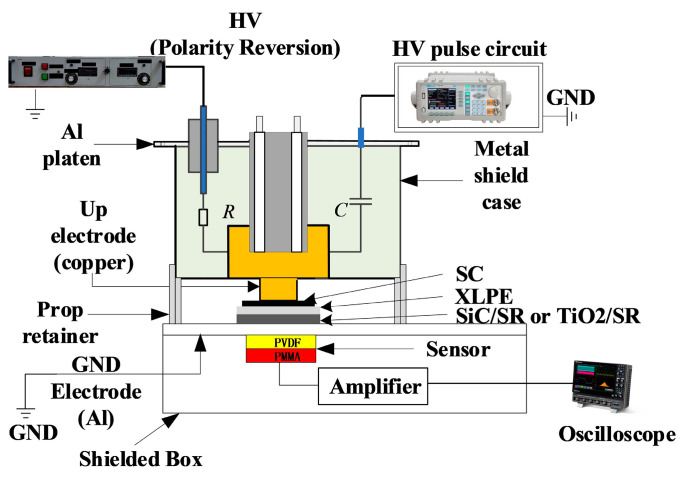
Schematic diagram of PEA space charge measurement platform.

A PEA measurement device capable of achieving polarity reversal was built, mainly consisting of four parts, as shown in Figure 5: high-voltage DC power supply, high-voltage pulse system, space charge measurement unit, and waveform automatic recording platform. On the basis of the original PEA measurement platform, the control of polarity reversal was achieved by adding a high-voltage DC power supply equipped with polarity reversal function.

**Figure 6:**
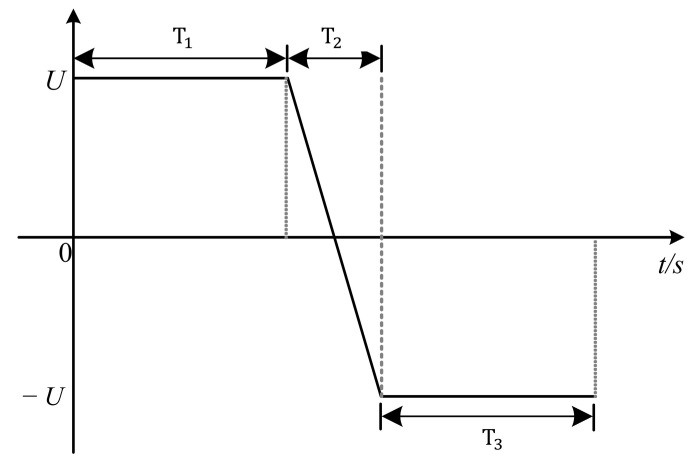
Polarity reversal voltage waveform applied during space charge testing.

For the space charge measurement of double-layer samples using the PEA method, the samples are laid flat in the following order: upper electrode, semi-conductive layer, XLPE, undoped pure SR, and the lower electrode. To ensure optimal contact between the layers, the media are coated with a thin layer of silicone oil. The average field strength of the applied polarization electric field is selected as 20 kV/mm, and the thickness of XLPE and nano-doped SR thin film samples is measured with a micrometer, which allows for precise determination of required polarization voltage U. The stages of applying DC voltage are divided into three stages: T_1_, T_2_, and T_3_. T_1_ is the polarization time before reversal, T_2_ is the polarity reversal process, T_3_ is the polarization time after reversal, and the applied voltage waveform is shown in Figure 6. During the T_1_ and T_3_ stages, the waveform signals on the oscilloscope at 1 min, 60 min, and 180 min after the completion of pressurization (0 s of T_1_) and the end of voltage reversal (0 s of T_3_) were recorded, respectively. The selection of polarity reversal time T_2_ refers to GB/T 31489.1-2015 with rated voltages of 500 kV, and the reversal time should be controlled within 120 s. In this experiment, T_2_ is taken as 30 s.

### 2.4. Breakdown Testing Platform and Testing Methods

**Figure 7:**
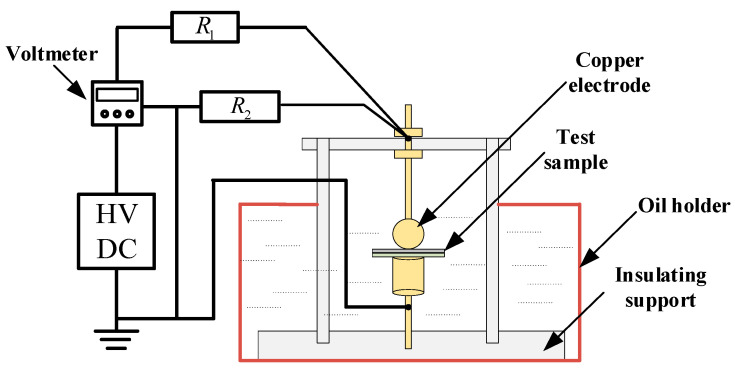
Diagram of breakdown test device for sheet-like specimens.

The breakdown platform in our experiments is designed in accordance with the International Electrotechnical Commission IEC 60243, which specifies the electrical strength test methods of solid insulation materials. The platform mainly consists of four parts: electrode system, oil tank, DC high-voltage source (capable of achieving polarity reversal function), protection, and measurement device, as shown in Figure 7.

**Figure 8:**
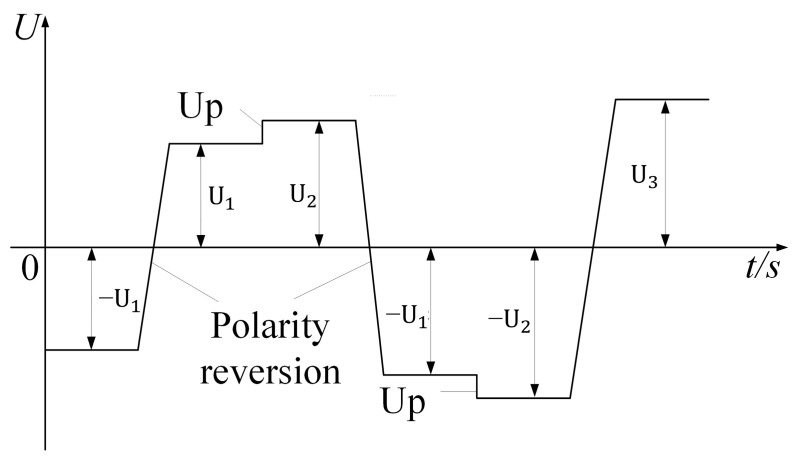
Polarity reversal voltage waveform applied during breakdown test.

In Figure 7, R_1_ and R_2_ are protective resistors, and the equipment parameters are shown in Table 2:

The DC breakdown follows the “Fast Boost Test Standard” in GB/T 1408.1-2016. The test involves a voltage ramp rate of 500 V/s, starting from 0 and boosting until the sample breakdown. The breakdown test process under polarity reversal voltage is shown in Figure 8. Firstly, a DC voltage was applied to the double-layer insulation sample, with a preloading time of 10 min to allow for a stable accumulation of space charges inside the sample. The voltage polarity reversal time is set to 30 s. If the sample does not experience breakdown after one complete polarity reversal, the voltage is slightly increased by 1 kV for each subsequent test. This process continues until sample breakdown. During the experiment, room temperature conditions were maintained. To reduce errors, each type of sample underwent 10 breakdown tests with the breakdown voltage recorded. The thickness of each breakdown point was measured using the micrometer. Finally, the breakdown field strength of the sample was calculated by dividing the applied voltage by the total thickness of the double-layer composite sample.

U_1_ is the set voltage for the first breakdown experiment, and U_2_ is the set voltage raised after the failure of the first breakdown experiment.

## 3. Space Charge Distribution under Polarity Reversal

**Figure 9:**
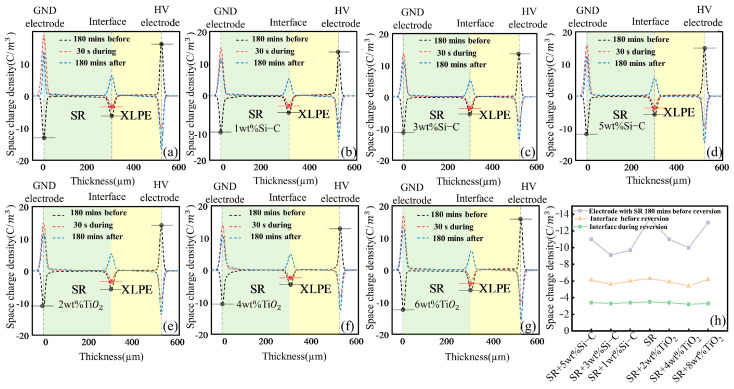
Space charge distribution under polarity reversal. (**a**) Pure SR/XLPE bilayer samples; (**b**) 1 wt% SiC-doped SR/XLPE bilayer samples; (**c**) 3 wt% SiC-doped SR/XLPE bilayer samples; (**d**) 5 wt% SiC-doped SR/XLPE bilayer samples; (**e**) 2 wt% TiO_2_-doped SR/XLPE bilayer samples; (**f**) 4 wt% TiO_2_-doped SR/XLPE bilayer samples; (**g**) 8 wt% TiO_2_-doped SR/XLPE bilayer samples. (**h**) Space charge density of different samples at the SR electrode interface inversion during different times.

The space charge distribution of nanocomposite SR/XLPE bilayer samples with different doping concentrations, both during and after voltage polarity reversal, is shown in Figure 9.

By comparing the space charge distribution before and after polarity reversal in Figure 9, it was found that the nanocomposite particle did not alter the general trend of the space charge density curve. However, doping particles with different concentrations significantly affect the amplitude and rate of changes in space charge distribution. Figure 9h shows the space charge density of different samples at the SR ground electrode and the interface before and after inversion. From the changes in space charge density at the SR before polarity reversal, it can be seen that doping SiC and TiO_2_ significantly reduces the space charge density at the ground electrode at the SR, mitigating the phenomenon of charge accumulation. The space charge density at the ground electrode of undoped SR is 13.5 C/m^3^, while the space charge density at the SR electrode doped with 3 wt% SiC and 4 wt% TiO_2_ is 9.1 C/m^3^ and 10 C/m^3^, reducing the accumulation of space charge by 32.6% and 25.9%, respectively.

From Figure 9h, it can be observed that the space charge at the interface decreased with the doping of SiC and TiO_2_, the moment before and after polarity reversal at the interface. The peak charge density at the undoped SR/XLPE interface before inversion was 6.3 C/m^3^. In contrast, the space charge density was reduced to 5.6 C/m^3^ for samples doped with 3 wt% SiC and 5.4 C/m^3^ for those doped with 4 wt% TiO_2_. After the 30 s reversal at the interface, it can be seen that the space charge density at the undoped SR interface decreased to 3.5 C/m^3^. In comparison, the space charge density at the interface of samples doped with 3 wt% SiC and 4 wt% TiO_2_ decreased to 3.3 C/m^3^ and 3.2 C/m^3^, respectively.

The changes in space charge density of different samples before and after inversion at the interface are listed in Table 3.

The peak charge density at the undoped SR/XLPE interface is 2.8 C/m^3^, both before and after inversion, which is higher than all the doped SR samples. In these two cases, the decrease in interface charge accumulation upon completing polarity reversal is more constrained in comparison to undoped SR, indicating a more pronounced “hysteresis effect”. That means that the time required for the space charge at the interface transition from its original state to a new steady state increases as the doping concentration rise from 0 wt% to “optimal concentration”. For samples doped with 3 wt% SiC and 4 wt% TiO_2_, the time required to reach a stable state is the longest. However, when the doping concentration exceeds these levels, the time needed for the accumulation of interface charges is shorter. This is because the interface charges are formed through the migration of free charges which then accumulate at the interface. However, these migrating charges are more readily trapped by internal traps within the sample. The charge transfer process initially requires overcoming trap constraints, which leads to a much lower rate of dissipation and migration compared to polarity reversal itself. The introduction of nanoparticles reduces the accumulation of charges both at the electrode and interface under a steady-state electric field during the reversal. Moreover, the presence of deep traps slows down the polarity change of interface charges, further influencing the reversal process.

The accumulation of space charges can cause local distortion of the electric field. The electric field superimposed by space charges follows a Poisson distribution [15]. Based on the density of space charges, the distribution of electric field E(x) in the sample can be calculated using the corresponding formula, as shown in Equation (1):

(1) $$E(x)=\int_{0}^{x}\frac{\rho (x)}{\varepsilon }dx,0\leq x\leq d$$

where E(x) is the electric field strength (kV/mm), ρ(x) is the space charge density (C/m^3^) at the thickness direction ×, ε is the dielectric constant of the insulation material.

**Figure 10:**
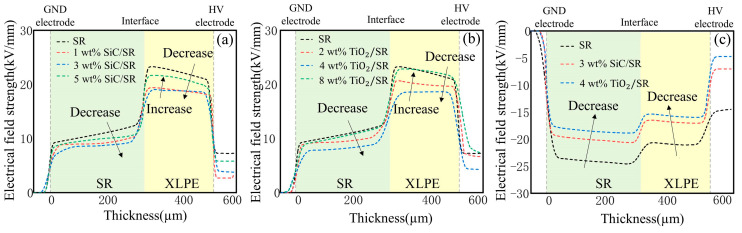
Electric field distribution of nanocomposite SR/XLPE bilayer samples with different doping concentrations. (**a**) Electric field distribution of SiC-SR/XLPE double-layer samples with different doping concentrations; (**b**) electric field distribution of TiO_2_-SR/XLPE double-layer samples with different doping concentrations; (**c**) comparison of 30 s reversal end electric field distribution between 3 wt% SiC-SR/XLPE double-layer samples and 4 wt% TiO_2_-SR/XLPE double-layer samples.

Based on the space charge measurement results and electric field calculation formula mentioned above, the electric field distribution of SiC and TiO_2_ nanocomposite SR/XLPE double-layer samples with different doping concentrations, both before and after voltage polarity reversal, is plotted as shown in Figure 10:

The accumulation of interface charges can cause serious distortion in the electric field distribution within the double-layer sample. However, the doping of nanoparticles effectively reduces the accumulation of these interface charges. As shown in Figure 10a,b, the introduction of 3 wt% SiC and 4 wt% TiO_2_ into SR can effectively reduce the distortion of the electric field within the double-layer medium. However, exceeding this concentration leads to an increase in the overall electric field distribution within the sample. Figure 10c shows the electric field distribution after polarity reversal. After the voltage polarity reversal is completed, the SR side sample experiences the maximum distorted electric field. The maximum field strength of the undoped SR sample reaches 24.56 kV/mm, whereas the maximum field strength of the 3 wt% SiC-SR sample is 22.08 kV/mm, and for the 4 wt% TiO_2_-SR sample, it is 20.94 kV/mm, representing a decrease of 10.01% and 14.74%, respectively. At doping concentration of 3 wt% for SiC-SR and 4 wt% for TiO_2_-SR, both types of nanoparticles not only significantly inhibit the accumulation of space charges inside the double-layer medium, but also effectively reduce the maximum distorted electric field that may occur in the SR sample.

## 4. Macroscopic Electrical Properties under Polarity Reversal

**Figure 11:**
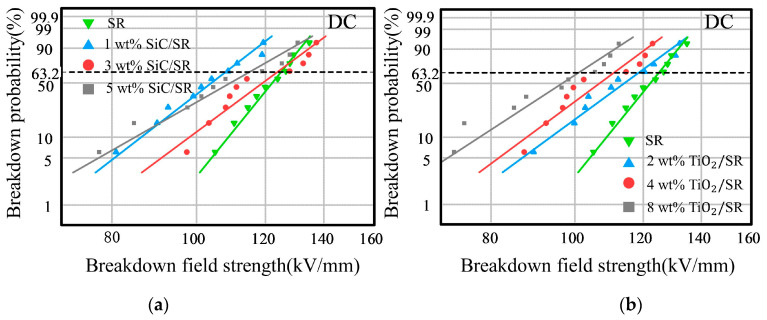
Weibull distribution of DC breakdown field strength in different nanocomposite materials. (**a**) Weibull distribution of DC breakdown field strength in nanocomposite SiC-SR. (**b**) Weibull distribution of DC breakdown field strength in nanocomposite TiO_2_-SR.

The conditions of polarity reversal and the doping of nanoparticles indeed influence the accumulation of the space charge at the interface of the double-layer dielectric, which in turn affects the degree of electric field distortion inside the insulation. The direct manifestation of this phenomenon at the macro level is the breakdown field strength. Figure 11 shows the Weibull distribution of DC breakdown. Table 4 and Table 5 show the characteristic breakdown field strength and shape parameters under DC.

From Figure 11, it can be observed that, compared with the DC breakdown field strength of undoped pure SR, the DC breakdown field strength of nanocomposite SR samples with different concentrations shows a noticeable decrease. Moreover, TiO_2_/SR nanocomposites with different concentrations showed a clear trend of “the higher the doping concentration of nanoparticles, the greater the decrease in breakdown field strength”. This occurs because SiC and TiO_2_ are semiconductor materials, and the higher concentrations reduce the average distance between the nanoparticles, increasing the likelihood of particle aggregation in the sample [16]. Furthermore, the presence of semiconductor materials can result in overlapping interaction areas between the interfaces of nanoparticles, forming a path similar to the conductive channels in polymer. This increased connectivity accelerates the migration rate of charge carriers. Consequently, it can lead to the easier formation of discharge channels inside the nanocomposite SR material, thereby increasing the probability of local breakdown, and reducing the overall voltage resistance performance [17,18,19,20].

However, for the nanocomposites with 3 wt% SiC/SR and 4 wt% TiO_2_/SR, which exhibit better inhibitory effects on the accumulation of interfacial charges, the breakdown field strengths are 123.73 kV/mm and 111.14 kV/mm, respectively. Compared to the breakdown field strength of pure SR, which is 125 kV/mm, the nanocomposite SR materials only exhibit a slight decrease. Therefore, under typical operating conditions of DC cables (field strength within 20 kV/mm), nanocomposite SR insulation materials can still provide a sufficient insulation margin [21,22,23,24,25].

**Figure 12:**
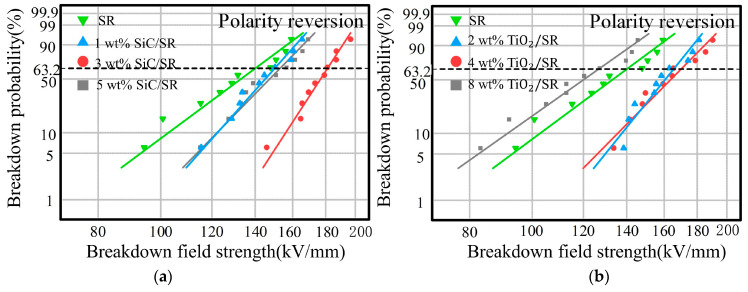
The 30 s polarity reversal breakdown characteristics of SR/XLPE double-layer dielectric with different doping: (**a**) 30 s polarity reversal breakdown characteristics of SiC-SR/XLPE double-layer dielectric; (**b**) 30 s polarity reversal breakdown characteristics of TiO_2_-SR/XLPE double-layer dielectric.

Figure 12 shows the Weibull distribution of polarity reversal, and Table 6 and Table 7 show the characteristic breakdown field strength and shape parameters.

In Figure 12, the breakdown field strength of the double-layer sample composed of SR and XLPE doped with nano-SiC shows an initial increase followed by a decrease as the nanoparticle concentration increases under the polarity reversal condition. Notably, the breakdown field strength is significantly higher than that of the sample without doping [26,27,28]. When the concentration of SiC particles reaches 3 wt%, the breakdown field strength of the double-layer sample peaks at 181.07 kV/mm. This represents a significant improvement of 29.43% compared to the 139.89 kV/mm breakdown field strength of undoped SR/XLPE under the same conditions. For the double-layer composite insulation composed of XLPE and SR doped with TiO_2_ particles, the breakdown field strength increases with doping concentration from 2 wt% to 4 wt%, reaching 169.8 kV/mm, which is a 21.38% improvement compared to pure SR. However, when the concentration of nano-TiO_2_ particles exceeds 8 wt%, the breakdown field strength significantly decreases to 126.52 kV/mm. This phenomenon corresponds to the maximum distortion of the electric field observed on the SR side during the polarity reversal process, as shown in Figure 10c. The maximum distortion of the electric field of 4 wt% TiO_2_ is the smallest. A smaller distortion of the electrical field requires a higher applied voltage to achieve the breakdown field strength of SR locally, resulting in a higher breakdown field strength of the double-layer sample [29,30].

## 5. Conclusions

This article establishes a correlation between composite nanoparticles and the electrical properties of XLPE/SR. It systematically studied the accumulation of space charge and local electric field distortion in the composite polymer structure under polarity reversal using nano-SiC and TiO_2_ of high-voltage DC cables. At the same time, the polarity reversal breakdown field strength of nanocomposite SR/XLPE double-layer dielectric was tested using a self-built breakdown test platform. Consequently, a correlation between the micro charge distribution characteristics and the macro insulation properties of the double-layer composite material was established. The main conclusions are as follows:

(1) Doping nanoparticles does not alter the overall trend of space charge distribution in double-layer media under polarity reversal conditions.
(2) Doping nanoparticles reduces the variation in space charge at the interface of the double-layer SR/XLPE under polarity reversal, making the “hysteresis effect” more pronounced. Specifically, the time required for space charge to accumulate at the interface after polarity reversal is shorter.
(3) Within the doping concentration limits, increased doping concentration leads to a decrease in interface charge accumulation. SiC particles at 3 wt% exhibit a significant inhibitory effect on interface charge accumulation, while the TiO_2_ at 4 wt% concentration effectively reduces the maximum distorted electric field intensity, which correlates with enhanced macroscopic breakdown strength.
(4) Doping nanoparticles improved the breakdown strength of the double-layer SR/XLPE, with notable improvements under polarity reversal conditions. Specifically, doping concentration with 4 wt% TiO_2_ increased the breakdown strength of the double-layer medium under polarity reversal by 21.38%.

## Figures and Tables

**Table 1:** Zeta potential of different SiC−SR/TiO_2_−SR samples.

Sample Type	Undoped Pure SR	1 wt% SiC-SR	3 wt% SiC-SR	5 wt% SiC-SR
Zeta potential (mV)	52	51	48	42
Sample type	Undoped pure SR	2 wt% TiO_2_-SR	4 wt% TiO_2_-SR	8 wt% TiO_2_-SR
Zeta potential (mV)	44	41	40	39

**Table 2:** Parameters of breakdown testing device.

Accessory	Type
DC high-voltage source	BGHX-20 kV
protective resistor	100 MΩ film resistor
oscilloscope	Lecroy HDO6054
pulse signal source	RIGOL2017B

**Table 3:** Changes in peak interface charge density of different SiC−SR/TiO_2_−SR samples over the same time period.

Sample Type	Undoped Pure SR	1 wt% SiC−SR	3 wt% SiC−SR	5 wt% SiC−SR
peak variation (C/m^3^)	2.8	2.6	2.3	2.7
Sample type	Undoped pure SR	2 wt% TiO_2_−SR	4 wt% TiO_2_−SR	8 wt% TiO_2_−SR
peak variation (C/m^3^)	2.8	2.5	2.2	2.9

**Table 4:** Parameters and characteristic breakdown field strength of DC breakdown in nanocomposite SiC/SR materials.

Sample Type	Undoped Pure SR	1 wt% SiC-SR	3 wt% SiC-SR	5 wt% SiC-SR
Shape parameter β	16.21	10.14	9.72	7.47
$\mathrm{External}\;\mathrm{application}\;\mathrm{characteristic}\;\mathrm{breakdown}\;\mathrm{field}\;\mathrm{strength}\;E_{0}$ (kV/mm)	125	107.89	123.73	114.91

**Table 5:** Weibull parameter in nanocomposite TiO_2_/SR materials.

Sample Type	Undoped Pure SR	2 wt% TiO_2_-SR	4 wt% TiO_2_-SR	8 wt% TiO_2_-SR
Shape parameter β	16.21	9.61	10.31	8.55
$\mathrm{External}\;\mathrm{application}\;\mathrm{characteristic}\;\mathrm{breakdown}\;\mathrm{field}\;\mathrm{strength}\;E_{0}$ (kV/mm)	125	118.43	111.14	100.89

**Table 6:** Parameters and characteristic breakdown field strength of 30 s polarity reversal breakdown in SiC−SR/XLPE double-layer dielectric.

Sample Type	Undoped Pure SR	1 wt% SiC−SR/XLPE	3 wt% SiC−SR/XLPE	5 wt% SiC−SR/XLPE
Shape parameter β	7.3	11.06	15.15	10.05
$\mathrm{External}\;\mathrm{application}\;\mathrm{characteristic}\;\mathrm{breakdown}\;\mathrm{field}\;\mathrm{strength}\;E_{0}$ (kV/mm)	139.89	149.99	181.07	152.86

**Table 7:** Parameters and characteristic breakdown field strength of 30 s polarity reversal breakdown in TiO_2_−SR/XLPE double-layer dielectric.

Sample Type	Undoped Pure SR	2 wt% TiO_2_-SR/XLPE	4 wt% TiO_2_-SR/XLPE	8 wt% TiO_2_-SR/XLPE
Shape parameter β	7.3	12.23	9.98	6.96
$\mathrm{External}\;\mathrm{application}\;\mathrm{characteristic}\;\mathrm{breakdown}\;\mathrm{field}\;\mathrm{strength}\;E_{0}$ (kV/mm)	139.89	165.38	169.80	126.52

## Data Availability

Data are contained within the article.
